# Retinoic acid related orphan receptor α is a genetic modifier that rescues retinal degeneration in a mouse model of Stargardt disease and Dry AMD

**DOI:** 10.1038/s41434-024-00455-z

**Published:** 2024-05-16

**Authors:** M. Akula, S. M. McNamee, Z. Love, N. Nasraty, N. P. M. Chan, M. Whalen, M. O. Avola, A. M. Olivares, B. D. Leehy, A. S. Jelcick, P. Singh, A. K. Upadhyay, D. F. Chen, N. B. Haider

**Affiliations:** 1 Schepens Eye Research Institute, Massachusetts Eye and Ear, Department of Ophthalmology, Harvard Medical School, Boston, MA USA; 2 Ocugen, Inc., Malvern, PA USA

**Keywords:** Genetics, Diseases

## Abstract

Degeneration of the macula is associated with several overlapping diseases including age-related macular degeneration (AMD) and Stargardt Disease (STGD). Mutations in ATP Binding Cassette Subfamily A Member 4 (*ABCA4*) are associated with late-onset dry AMD and early-onset STGD. Additionally, both forms of macular degeneration exhibit deposition of subretinal material and photoreceptor degeneration. Retinoic acid related orphan receptor α (*RORA*) regulates the AMD inflammation pathway that includes *ABCA4, CD59, C3* and *C5*. In this translational study, we examined the efficacy of *RORA* at attenuating retinal degeneration and improving the inflammatory response in *Abca4* knockout (*Abca4*^−/−^) mice. AAV5-*hRORA-*treated mice showed reduced deposits, restored CD59 expression and attenuated amyloid precursor protein (APP) expression compared with untreated eyes. This molecular rescue correlated with statistically significant improvement in photoreceptor function. This is the first study evaluating the impact of *RORA* modifier gene therapy on rescuing retinal degeneration. Our studies demonstrate efficacy of *RORA* in improving STGD and dry AMD-like disease.

## Introduction

Age related macular degeneration (AMD) is a complex retinal degenerative disease that is a leading cause of central vision loss in older adults. AMD is multigenic, also impacted by environmental influences and only naturally occurring in humans [1–8]. Dry AMD is the most common form, accounting for about 90% of AMD cases, and involves formation of deposits in the subretinal and sub-retinal pigment epithelium (RPE) spaces, damaging the macula, which is required for clear central vision [9]. While AMD is typically diagnosed after age 65, it can occur as early as age 45 [refs. 10–12]. In contrast, Stargardt Disease (STGD), with an incidence rate of 1 in 8 000–10 000, is a juvenile form of macular degeneration that includes early onset vision loss starting as early as 10 years of age [13–18]. Vision loss is typically observed in the first decade of life; however, some patients do not begin to lose vision until adulthood [15, 19]. STGD results from a build-up of fatty material on the macula of the retina, and causes retinal degeneration and vision loss. The most common symptoms of STGD are a slow, bilateral loss of central vision, dark or hazy spots in the visual center [20], sensitivity to light [21], longer time to dark adaptation [22–24] and color blindness [25]. STGD patients also show complement system dysregulation in the RPE [26]. Recently, two new drugs, pegcetacoplan (Syfovre) [27] and avacincaptad pegol (Izervay) [28], were FDA approved to treat geographic atrophy secondary to dry AMD. Both of these drugs target specific genes in the complement and inflammatory pathways. Pegcetacoplan is a complement C3 inhibitor requiring injection at least every other month; however, patients are at risk of developing exudative AMD following treatment (8.9%) compared to sham-treated eyes (1.2%) [ref. 27]. Similarly, avacincaptad pegol targeting complement C5 requires monthly injections, and patients treated with this drug had a 4-9.6% chance of developing choroidal neovascularization compared to 2.7% for sham-treated eyes [29]. Thus, there is great value in continuing to develop and test novel targets that can prevent or treat different pathways for dry AMD and STGD.

STGD and dry AMD have significant overlap in the clinical, molecular, and genetic phenotypes such as formation of deposits in the subretinal region, RPE cell damage, perturbations in the complement pathway and ATP Binding Cassette Subfamily A Member 4 (*ABCA4*) variants associated with each disease [13, 15–17, 26, 30–38]. Prior publications show that heterozygous *ABCA4* variants are associated with AMD, with one study suggesting that *ABCA4* mutations may be dominantly inherited in AMD [30–32, 39]. In contrast, the most common cause of STGD results from autosomal recessive mutations in the *ABCA4* gene [40–42]. Mutations in the *ABCA4* gene lead to faulty processing and transportation of all-trans retinaldehyde from photoreceptors to the RPE. Mutations in *ABCA4* cause accumulation of N-retinylidene-N-retinyl-ethanolamine (A2E), the main fluorophore found in lipofuscin forming deposits in the RPE [43]. STGD typically presents with progressive bilateral loss of central vision paired with the appearance of subretinal lipofuscin flecks in the macula [44]. *ABCA4*-associated retinopathies manifesting early in life progress more rapidly, while a later age of onset is associated with a milder prognosis and can be misdiagnosed as age-related macular degeneration (AMD) [6, 42, 45].

**Fig. 1:**
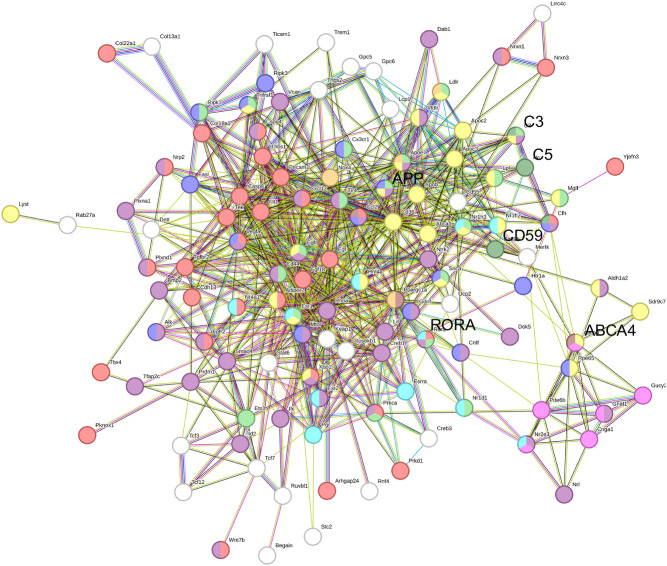
*RORA*-regulated gene pathways. String pathway analysis demonstrates the relationship between ABCA4, RORA and CD59 in the inflammatory response and complement gene pathways regulated by *RORA*. Each node depicts a protein encoded by a gene, and each line represents an association between proteins. Node colors represent specific *RORA*-regulated pathways: angiogenesis is depicted in red, neuroprotection using indigo, the inflammatory response in light green, the complement cascade in dark green, lipofuscin formation in brown, oxidative stress in orange, lipid metabolism in yellow, phototransduction in pink and neurogenesis in mauve. Known interactions are shown using blue (curated databases), bright green (proteins mentioned in publications) or pink lines (experimentally determined proteins), while predicted interactions are shown in green (proteins in close proximity) or dark blue lines (co-occurring proteins).

Retinoic acid related orphan receptor α (*RORA*) is a nuclear hormone receptor (NHR) that regulates several homeostatic pathways in the retina including the lipid metabolism, oxidative stress and inflammation pathways [46–48]. Prior results demonstrated that *RORA* is linked with AMD and is a known regulator of multiple AMD genes [2, 48, 49]. Furthermore, pathway analysis by our lab shows that *RORA* regulates the inflammatory response pathway that includes *ABCA4* and *CD59* (Fig. 1). CD59 is an inhibitor of membrane attack complex (MAC) which is assembled with the help of complement C3 and C5 proteins, both of which are implicated in STGD and AMD disease [26, 35, 36]. Thus, *RORA* has the potential to be a more powerful therapeutic as a regulator of AMD-associated genes. In the current study, we evaluate AAV5-*hRORA* as a modifier gene therapy for macular degeneration using the *Abca4*^−/−^ mouse displaying both the clinical and molecular phenotypes of dry AMD and STGD [22, 33, 43, 50].

## Materials and methods

### Animal use and ethics

This study was performed in accordance with the Guide for the Care and Use of Laboratory Animals of the National Institute of Health and the Association for Research in Vision and Ophthalmology Statement for the Use of Animals in Ophthalmic and Vision Research. *Abca4*^−/−^ (*Abca4*^*tm1Ght*^*/*J, Stock #023725, Jackson Laboratories, Bar Harbor, ME, USA) mice and 129S1 (129S1/SvImJ, Stock #002448, Jackson Laboratories) wild type (WT) animals were housed under standard conditions at a temperature between 20 and 25 °C in a 12-h light and 12-h dark cycle. The Schepens Eye Research Institute Animal Care and Use Committee approved all animals and procedures used for this study (Protocol Number: 2020N000178) in compliance with the regulations of the Animal Welfare Act.

### Scientific rigor and reproducibility

A power calculation was conducted using G*Power 3.1 software analysis for estimating the sample size required for each type of analysis. Using the means and standard deviation defined by previously published studies, a minimum of five animals per experimental group were used to provide over 90% power and a 30% difference with a significance level set at 0.05. Every procedure was carried out using a standardized protocol. This study was conducted by several trained personnel in a double blinded and randomized way in order to prevent bias. Five biological replicates were used at a minimum for each dose and time point to reach statistical significance. Animals with unresolved surgical trauma, premature unintended death or cataracts, or data resulting from technical error were excluded from the study. Less than 10% of the 60 total treated experimental animals were excluded from all analyses based on these criteria. An equal number of males and females were used, as sex is not a biological variable in this study.

### Statistics

The differences in the mean blue autofluorescence (BAF) levels, scotopic b-wave amplitudes, recovery of a-wave amplitudes, the number of cell layers in the ONL, and the mean CD59 and RORA immunolabeling fluorescence intensity levels for each dose at each time point were analyzed using a one or two way analysis of variance (ANOVA) and Tukey’s post-hoc analysis for multiple comparisons using GraphPad Prism 9.0. All data are presented as mean ± standard error of mean (SEM).

### Test article

The AAV5-*hRORA* drug product and formulation buffer consisting of 10 mM sodium phosphate, 180 mM sodium chloride, and 0.001% Poloxamer 188 (PF-68, Landrau Scientific Innovations, Leominster, MA, USA) at a pH of 7.3 ± 0.5 were supplied by Ocugen Inc. (Malvern, PA, USA). The intended doses (1 × 10^8^, 1 × 10^9^, and 4 × 10^9^ vg/eye) were prepared for treatment and randomized. *AAV5* was selected as a viral vector based on its safety and transduction efficiency in photoreceptors and RPE cells [51].

### Subretinal injections

*Abca4*^−/−^ mice were given a subretinal injection with one of the three different doses of AAV5-*hRORA*. As a control, B6 mice were given a subretinal injection with AAV5-*hRORA*-GFP at 1 month (1M) of age. Mice were anesthetized with ketamine/xylazine. An incision was made in the sclera adjacent to the cornea using a 30 G needle. Slight resistance indicated that the Bruch’s membrane had been breached. 0.5 µl of the therapeutic was administered using a Hamilton syringe with a blunt cannula (33 G) where the cut was made using a 30 G needle, into the subretinal space. Only the right eye was treated, and the left eye was either left uninjected, or an incision was made (mock) or injected with the formulation buffer (mock + buffer).

### Genotyping

DNA was isolated from mouse tail biopsy samples by lysing the samples using sodium hydroxide method. DNA samples were amplified using the following primers: the common forward primer, AGG AGA AGC AAT CAA ATC AGG A; the WT reverse, GAA GAT GCT CTG GAT ATC TCT GC; and the mutant reverse primer, TGA GTA GGT GTC ATT CTA TTC TGG. The WT reverse primer amplifies a 4 kb region from the promoter and exon 1 of the *Abca4* gene, while the mutant reverse primer amplifies the neo cassette replacing this region [35, 52]. For PCR amplification, approximately 50 ng of DNA was used in a 10 *μ*L reaction consisting of 10x buffer that included MgCl_2_, 40 *m*M of dNTP mix, 10 *μ*M of forward and reverse primer, and 5 U/mL of AmpliTaq DNA polymerase. Reactions were denatured at 94 °C for 2 min followed by 10 cycles at 94 °C for 30 s, 65 °C for 30 s with a reduction of 0.5 °C per cycle and 72 °C for 30 s, and then 28 cycles at 94 °C for 15 s, 60 °C for 30 s, 72 °C for 30 s and a final extension at 72 °C for 5 min. Amplified samples were separated by size using a 2% agarose gel and imaged under UV light after ethidium bromide staining. The WT amplicon is 123 base pairs long, and the mutant amplicon is 160 base pairs [35].

### Blue light autofluorescence (BAF)

Fundus BAF imaging was used to measure the presence of lipofuscin as previously described [22], to test whether AAV5-*hRORA* treatment is able to resolve this phenotype. Animals were anesthetized with ketamine/xylazine by intraperitoneal (IP) injection, and 1% tropicamide was used for pupil dilation. BAF images were captured in response to light at 488 nm using a confocal scanning laser ophthalmoscope machine at the same light intensity, exposure and acquisition settings for all mice (Spectralis, Heidelberg, Germany).

### Electroretinogram (ERG) analysis

#### Full field ERG

Scotopic and photopic ERGs were acquired as previously described [46, 53, 54] to measure retinal function (Diagnosys LLC, Lowell, MA, USA). Mice were dark adapted for at least 4 h to examine rod function through scotopic responses, and anesthetized via an IP injection of ketamine and xylazine diluted with saline. Gold loop electrodes were placed on the apex of the cornea for each eye. Silver needle electrodes were inserted subcutaneously in the bridge of the nose as a reference and in the base of the tail as a ground electrode. Responses to short wavelength flashes of light at 10 ms were acquired at 0.4 log intensity increments starting at an intensity of 0.000249 cd·s/m^2^ up to 24.1 cd·s/m^2^. Light-adapted responses were obtained similarly after 7 min of exposure to background light starting with an intensity of 0.1 cd·s/m^2^ up to 25.6 cd·s/m^2^ at 0.4 log intensity increments. The peak b-wave amplitude of AAV5-*hRORA*-treated *Abca4*^−/−^ mice was averaged and graphed in comparison to peak amplitude values from WT and untreated *Abca4*^−/−^ mice. The b-wave amplitude was also plotted over increasing log step intensity values in both treated and untreated *Abca4*^−/−^ mice compared with WT mice.

#### Recovery after photobleaching ERG

Mice were anesthetized using an IP administration of ketamine and xylazine mixed with saline, and were given another IP injection of ketamine at half of the original concentration when animals showed signs of awakening. After initial anesthesia, pupils were dilated and the baseline ERG waveform was acquired at 25 cd⋅s/m^2^. The retinas were subsequently photobleached using white light at 400 lux for 5 min, after which responses were recorded every 5 min at 25 cd⋅s/m^2^ for 1 h [22, 23, 55]. The peak percentage recovery of all analyzed eyes were normalized to the peak of the 129S1 control eyes, which was mathematically adjusted to 100%.

### Histology

Histological analysis was carried out as described previously [54, 56, 57]. Eyes were collected, a cautery mark was made at the dorsal orientation of the eye and fixed in either methanol/acetic acid or 4% paraformaldehyde for paraffin embedding. After embedding, samples were sectioned (5 µm/section) for hematoxylin and eosin (H&E) staining. Serial sections were collected at a similar depth in the central retina to reduce variability. Stained retinal sections were imaged on a DMI6000 Leica microscope (Wetzlar, Germany). The number of photoreceptor cell layers in the outer nuclear layer (ONL) were counted by a minimum of three double blinded personnel. The number of cell layers in the ONL was counted in three regions of the central retina at a similar retinal depth (~400 µm), and compared between untreated *Abca4*^−/−^ retinas and *Abca4*^−/−^ eyes treated with three doses of AAV5-*hRORA* [46].

### Immunohistochemistry (IHC)

IHC was performed as previously described [46]. Eyes collected for histology were also used for immunohistochemical analysis. Tissue sections of a similar retinal depth (~400 µm) were deparaffinized, rehydrated, and 2% blocking serum applied for 1 h at room temperature, followed by incubation in primary antibody overnight. The CD59 antibody conjugated to FITC (mouse monoclonal, ab180633, Abcam, Waltham, MA, USA), the amyloid precursor protein (APP) antibody (rabbit polyclonal, 36–6900, Thermo Fisher, Waltham, MA, USA) and the RPE65 antibody (mouse monoclonal, MA1-16578, Thermo Fisher, Waltham, MA, USA) were used at a 1:100 dilution, and an antibody raised against RORA (rabbit, 101401, Genscript, Piscataway, NJ, USA) was used at a 1:500 dilution. An Alexa Fluor goat anti-rabbit or anti-mouse secondary antibody (A-21428, Thermo Fisher, Waltham, USA) was used to probe for the APP antibody or the RPE65 antibody, respectively. WT 129S1 tissue was used as a reference for all doses. IHC slides were imaged on the DMI6000 Leica microscope. The mean fluorescence intensity of CD59 and RORA in the inner/outer segments (IS/OS) and RPE layers of WT, and untreated and AAV5-*hRORA*-treated *Abca4*^−/−^ retinas was evaluated using ImageJ [58] by manually selecting the IS/OS or RPE regions and measuring the mean gray levels.

### Chromatin immunoprecipitation sequencing (ChIP-seq)

Putative target genes were analyzed for the *Rora* nuclear receptor binding site, (AGT)(TA)(AT)(TA)C(AT)AGGTCA, as previously described [48]. Binding sites were chosen at a maximum of 100 kilobases (kb) upstream of the start site of intron 1 for each gene. Retinas were dissected out from C57Bl/6 J mice, homogenized and fixed in 37% formaldehyde. The chromatin was sonicated to an average length of ~600 bp, followed by immunoprecipitation using 4 ug of a RORA antibody (Thermo Fisher, PA1-812, Waltham, MA, USA) overnight at 4 °C. Samples were then incubated with Protein G dynabeads (10003D, Invitrogen, Waltham, MA, USA), and the beads were collected by centrifuging the samples at 10,000 rpm for 3 min, and washed in wash buffer and TE buffer. The protein-DNA complexes were eluted from the beads, and the cross links were removed using 200 mM NaCl. The chromatin DNA then underwent purification, and was sent for ChIP-seq (Harvard Bauer Core, Center for Systems Biology) and the data analyzed (Harvard Bioinformatics Core).

### String pathway analysis

ChIP-seq data was further compared with AMD-linked pathways, including angiogenesis, lipid metabolism, inflammatory response, complement system and oxidative stress using String (version 10) [ref. 59]. Networks were created based on the connectivity between the genes. Each node depicts a protein encoded by the corresponding gene, and each line represents a functional or physical association between proteins. Known interactions are given in blue (curated databases), bright green (proteins mentioned together in publications) or pink lines (experimentally determined proteins), while predicted interactions are depicted in green (proteins in close proximity) or dark blue lines (co-occurring proteins).

## Results

### Reduced BAF levels in AAV5-*hRORA*-treated *Abca4*^−/−^ retinas

*RORA* is a NHR that regulates numerous AMD-linked pathways, including angiogenesis, lipid metabolism and the inflammatory response pathways (Fig. 1). In particular, *RORA* regulates the inflammatory response and complement pathways that includes CD59, C3 and C5. Here, we evaluated *RORA* as a potent, broad-spectrum modifier gene therapy for macular degeneration using the *Abca4*^−/−^ mouse model. Distribution of RORA in the fundus was assessed by subretinal delivery of AAV5-*hRORA* tagged with GFP (AAV5-*hRORA*-GFP) into B6 mice. GFP was evaluated using BAF imaging, as this detects the presence of fluorescent particles excited by blue light at 488 nm, including GFP (Fig. S1). AAV5-*hRORA*-GFP was distributed throughout the fundus compared with the untreated B6 fundus. Moreover, no apparent adverse effects of treatment were observed on the fundus. Immunohistochemical evaluation of RORA was performed to determine expression in the retinal layers using WT (129S1) and *Abca4*^−/−^ tissue sections, which showed very little expression in untreated photoreceptor IS/OS, and RPE tissue compared with the WT strain (Fig. S2A, B). *Abca4*^−/−^ eyes treated with AAV5-*hRORA* showed a significant elevation in RORA immunolabeling fluorescence intensity in the IS/OS (Fig. S2B, *p* < 0.05 to 0.0001) and RPE (*p* < 0.01 to 0.0001) at all three doses compared with untreated *Abca4*^−/−^ eyes.

**Fig. 2:**
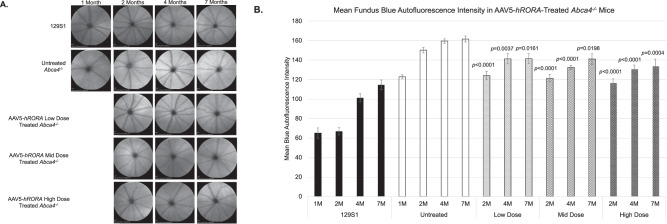
Reduced blue autofluorescence (BAF) (488 nm) in the fundus of AAV5-*hRORA* treated *Abca4*^−/−^ mice. **A** Fundus BAF images show increased AF levels in untreated *Abca4*^−/−^ mice compared with 129S1 (control) retinas, and a reduction in BAF in *Abca4*^−/−^ eyes treated with AAV5-*hRORA* at all three doses. **B** Quantification of autofluorescence as measured by mean gray levels shows a reduction in the BAF levels of *Abca4*^−/−^ eyes treated with either a low, mid or high dose of AAV5-*hRORA* at 1 month (1M), 3M and 6M post-treatment compared with untreated eyes at all ages studied. Mean ± SEM, *n* ≥ 5 biological replicates.

AAV5-*hRORA*-treated *Abca4*^−/−^ mice were next evaluated using BAF fundus imaging to determine if *RORA* can rescue the dry AMD-like phenotype of lipofuscin deposits observed in *Abca4*^−/−^ mice [22, 50]. The effect of treatment was longitudinally assessed at 1M, 3M, and 6M post-treatment until the intermediate disease stage. Animals treated with AAV5-*hRORA* showed a reduction in the presence of subretinal deposits that was consistent across doses and ages examined (Fig. 2). Quantification of the levels showed that all three doses elicited a statistically significant reduction in BAF levels that is reflective of the amount of lipofuscin (Fig. 2B; *p* < 0.05 to 0.0001).

### Improved photoreceptor function in AAV5-*hRORA*-treated *Abca4*^−/−^ mice

**Fig. 3:**
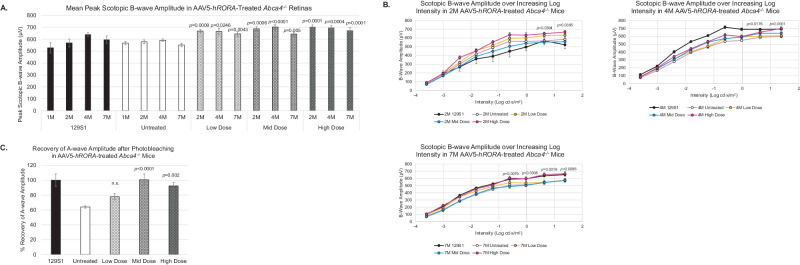
Increased scotopic b-wave amplitudes in AAV5-*hRORA* treated *Abca4*^−/−^ mice. **A** Untreated 1M and 2M *Abca4*^−/−^ mice display a similar peak scotopic b-wave amplitude as control 129S1 mice. At 2M, eyes treated with AAV5-*hRORA* show a slight increase in the peak scotopic b-wave amplitude. There is a similar increase in peak scotopic b-wave amplitude at 4M and 7M in eyes treated with AAV5-*hRORA* compared with untreated eyes. **B** A statistically significant increase in scotopic b-wave amplitude was observed in AAV5-*hRORA* high dose-treated *Abca4*^−/−^ retinas at the highest intensity steps compared with untreated eyes at all ages tested (*p* < 0.05 to 0.0001). **C** The peak percent recovery of the baseline a-wave amplitude after photobleaching for 5 min at 400 lux exhibits a statistically significant increase in mid and high dose treated eyes compared with untreated eyes. Mean ± SEM, *n* ≥ 5 biological replicates.

The effect of AAV5-*hRORA* treatment on photoreceptor function was subsequently evaluated in *Abca4*^−/−^ mice by using scotopic ERGs and testing recovery of photoreceptors after photobleaching. ERGs were performed up to 7M, as retinal degeneration phenotypes including lipofuscin deposits are apparent by this age [22]. The scotopic b-wave amplitudes were measured in AAV5-*hRORA*-treated *Abca4*^−/−^ animals to test the effect of AAV5-*hRORA* on photoreceptor function. ERG analysis of all three doses demonstrated a slight increase in peak scotopic b-wave amplitude in eyes treated with AAV5-*hRORA*. Prior studies on this mouse model show that the scotopic b-wave amplitude is within the normal range [22, 50]. However, high dose-treated *Abca4*^−/−^ eyes showed statistically significant improvement in the b-wave amplitude compared with untreated eyes (Fig. 3A, B; *p* < 0.05), and importantly, improvement was sustained until 7M of age (Fig. 3A, B). Moreover, there were no apparent adverse effects of AAV5-*hRORA* treatment on photoreceptor function.

Previous studies show that *Abca4*^−/−^ mice exhibit reduced recovery of scotopic a-wave amplitude after a photobleaching event when compared with control animals [23]. Analysis showed that AAV5-*hRORA*-treated eyes at the mid and high doses showed a statistically significant increase in peak percentage recovery of the baseline a-wave amplitude after photobleaching when compared with untreated eyes (Fig. 3C; *p* < 0.01 to 0.0001).

### No apparent adverse effect of AAV5-*hRORA* treatment on *Abca4*^−/−^ retinal morphology

**Fig. 4:**
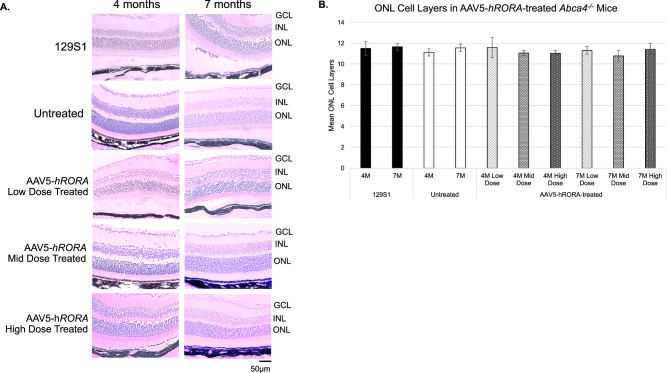
No adverse effects in *Abca4*^−/−^ retinas treated with AAV5-*hRORA.* **A** Histological analysis did not show a difference in the thickness of the retinal layers in the treated retinas compared with untreated eyes and the control background strain. **B** Quantification of the ONL layer number between untreated and AAV5-*hRORA*-treated *Abca4*^−/−^ mice showed no statistically significant change. GCL ganglion cell layer, INL inner nuclear layer, ONL outer nuclear layer; Mean ± SEM, *n* ≥ 5 biological replicates. Scale bars 50 µm.

H&E staining was performed to examine the effect of *RORA* treatment on retinal morphology and to assess potential adverse effects. Histological staining demonstrated no gross adverse effect of treatment in AAV5-*hRORA* treated knockout animals when compared with 129S1 mice and untreated *Abca4*^−/−^ mice (Fig. 4), which is consistent with the lack of adverse effects on the fundus in B6 eyes treated with AAV5-*hRORA*-GFP (Fig. S1), and absence of adverse effects on photoreceptor function in AAV5-*hRORA*-treated *Abca4*^−/−^ eyes (Fig. 3A, B). Normal cell topography was observed in both untreated and AAV5-*hRORA-*treated *Abca4*^−/−^ retinas (Fig. 4A). The number of cell layers in the ONL was counted, which showed no statistically significant difference between untreated *Abca4*^−/−^ eyes and *Abca4*^−/−^ eyes treated with the three doses of AAV5-*hRORA* (Fig. 4B).

### Molecular rescue of the inflammatory response in AAV5-*hRORA*-treated *Abca4*^−/−^ retinas

**Fig. 5:**
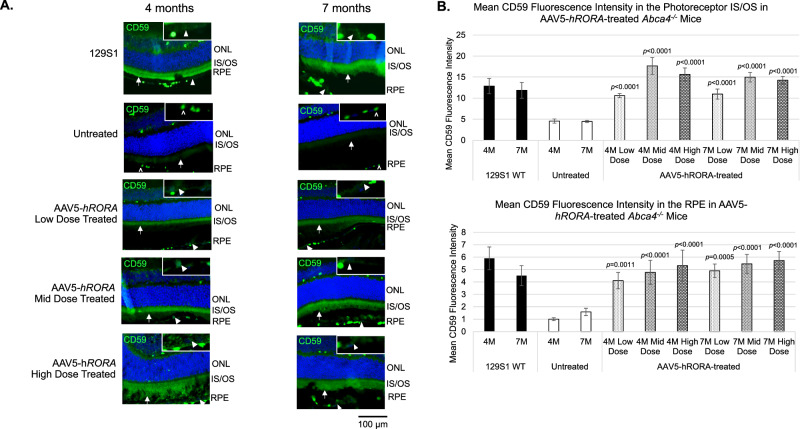
Restored expression of CD59 in *Abca4*^−/−^ mice treated with the low, mid and high doses of AAV5-*hRORA.* **A** CD59 expression is observed in the inner/outer segments (IS, OS) (white arrows) of the photoreceptors and the retinal pigment epithelium (RPE) (insets, closed white arrowheads) in 4M and 7M 129S1 control retinas that is lacking in the untreated *Abca4*^−/−^ IS/OS region and RPE. CD59 expression is restored in AAV5-*hRORA* treated *Abca4*^−/−^ eyes in the IS/OS region (white arrows) and the RPE (insets, closed white arrowheads) at all three doses. Open arrowheads indicate autofluorescence in blood vessels. **B** There is a statistically significant increase in CD59 mean fluorescence intensity in both the photoreceptor IS/OS region (*p* < 0.0001) and the RPE (*p* < 0.01 to 0.0001) in *Abca4*^−/−^ eyes treated with all doses of AAV5-*hRORA* compared with untreated *Abca4*^−/−^ eyes. Mean ± SEM; WT, *n* ≥ 3 biological replicates; *Abca4*^−/−^, *n* ≥ 5 biological replicates. Scale bars 100 µm.

**Fig. 6:**
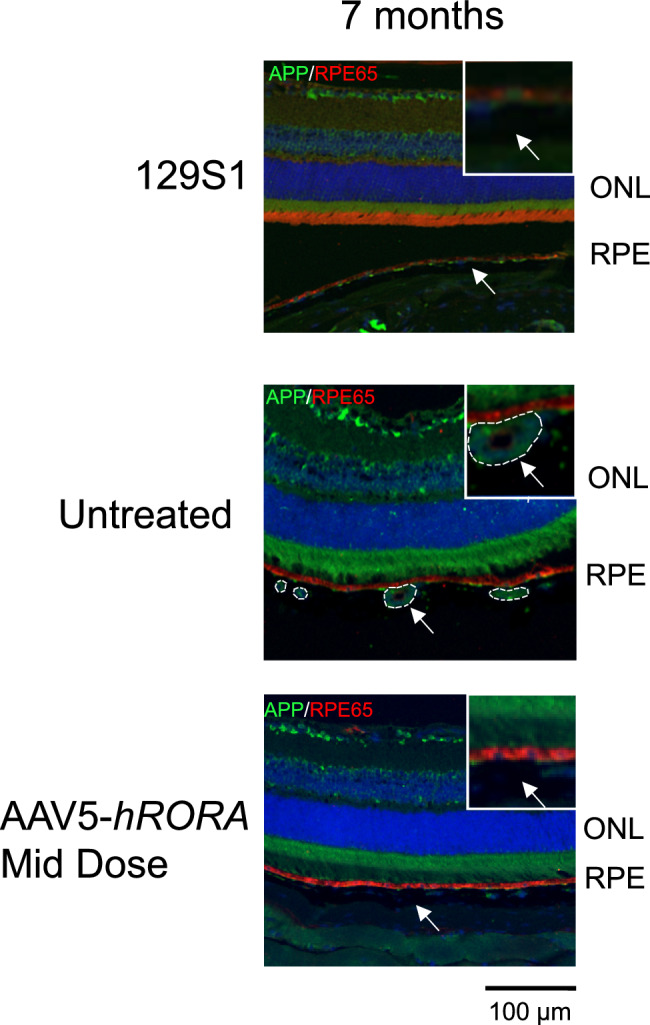
Reduced expression of amyloid precursor protein (APP) in AAV5-*hRORA*-treated *Abca4*^−/−^ mice. APP expression (green) was observed in the sub-RPE region of untreated *Abca4*^−/−^ eyes at 7M (2nd panel, outlined by dashed lines, arrows), while expression was attenuated in *Abca4*^−/−^ retinas treated with the mid dose of AAV5-*hRORA*. *n* = 5 biological replicates. Scale bars 100 µm.

*RORA* regulates the inflammatory response pathway that includes complement pathway genes and is implicated in both the STGD and dry AMD-like phenotypes [34–37, 47]. In particular, CD59, an inhibitor of the MAC, is downregulated in both diseases and is part of the *RORA*-regulated inflammatory response pathway that also includes *ABCA4* [33, 36, 37]. IHC was performed for CD59 to determine if AAV5-*hRORA* can restore CD59 expression in *Abca4*^−/−^ mice. We observed restoration of CD59 expression in AAV5-*hRORA* treated animals in the photoreceptor IS/OS and RPE (Fig. 5A). Moreover, there was a statistically significant increase in CD59 immunolabeling fluorescence intensity in the photoreceptor IS/OS (Fig. 5B, *p* < 0.0001) and the RPE (*p* < 0.01) of AAV5-*hRORA*-treated *Abca4*^−/−^ eyes compared with untreated eyes. Treatment with AAV5-*hRORA* not only improved CD59 expression, but the expression was comparable to the WT expression levels. Rescue of the molecular phenotype is consistent with rescue of clinical retinal degeneration phenotypes, including attenuated lipofuscin. Amyloid-β is an inflammatory component of deposits found in AMD that is derived from APP [60–63]. Consequently, IHC was performed for APP, with the data showing reduced expression of this protein in the sub-RPE region of treated retinas when compared with untreated *Abca4*^−/−^ eyes (Fig. 6).

## Discussion

Nuclear hormone receptors regulate numerous retinal homeostatic pathways. In particular, *RORA* is implicated in AMD and is a key regulator of pathways perturbed in AMD disease, such as the complement system including complement C3 and C5 that are targets of recent FDA-approved drugs for dry AMD [27, 28, 46, 47]. In this study we test the potential of three different doses of AAV5-*hRORA* as a modifier gene therapy to treat macular degeneration in *Abca4*^−/−^ mice, as it is a model for both early onset STGD and late onset dry AMD, and exhibits subretinal deposits and reduced retinal function, as well as perturbation of complement genes [22, 33, 34, 37, 64, 65].

Our data demonstrated rescue of STGD and dry AMD-like retinal degeneration across multiple clinical, functional and molecular measures in *Abca4*^−/−^ mice. BAF imaging showed clinical restoration reflecting a reduction in the level of lipofuscin and other deposits [43, 65, 66] at all time points in *Abca4*^−/−^ mice treated with AAV5-*hRORA*, with a more pronounced rescue at the mid and high doses. *Abca4* is an ATP-binding cassette transporter responsible for clearing the bisretinoid A2E, a toxic byproduct of the phototransduction cycle and the major fluorophore found in lipofuscin that emits autofluorescence picked up by BAF imaging [67]. A2E accumulation in the RPE prevents cholesterol efflux from RPE cells [68] and leads to RPE cell degeneration, eventually causing photoreceptor loss and functional deficits [23]. According to Zhang et al., vitamin A byproducts such as A2E may compete with vitamin A, even binding to RPE65 and retinoic acid receptor [69]. This could then inhibit opsins, interfering with the phototransduction cycle and leading to delayed dark adaptation [69]. The reduction in BAF levels reflecting lipofuscin deposition in *RORA*-treated *Abca4*^−/−^ retinas is likely responsible for the observed improvement in functional recovery of photoreceptors after exposure to high-intensity light. The scotopic b-wave amplitude in untreated *Abca4*^−/−^ eyes are within the normal range until a later age [22]; however, there is a statistically significant elevation in b-wave amplitude in high dose-treated eyes, demonstrating that AAV5-*hRORA* treatment improves retinal function. Importantly, this improvement in function was sustained at 7M, and there were no observed adverse effects of *RORA* treatment on photoreceptor function.

In addition to clinical and functional improvements, the current study also demonstrated rescue of the molecular phenotype in *Abca4*−^*/*^− mice. Restoration of CD59 expression in AAV5-*hRORA*-treated retinas suggests that the mechanism of action underpinning the observed functional and clinical improvements involves the complement pathway. A previous study showed that vitamin A byproducts, such as A2E, could interfere with CD59 recycling upon MAC activation, leading to its reduced expression in *Abca4*^−/−^ mice [37]. In the current study, regulation of the inflammatory response pathway by *RORA* is likely resulting in the observed restoration of CD59 expression, counteracting MAC activation. The attenuated inflammatory response could potentially lead to reduced presence of lipofuscin, as well as APP, the precursor for amyloid-β, a protein that increases inflammation and is normally found in human AMD deposits [60–63, 70, 71]. The APP antibody used in the current study potentially probes for both pathological amyloid-β, in addition to non-pathological APP. Expression of APP in the IS/OS observed in the current study could reflect accumulation of amyloid-β due to aging as demonstrated in previous animal studies [72–74]. On the other hand, APP expression observed in the inner retinal layers is likely because of its role in normal function in these layers [75].

*RORA* regulates numerous pathways associated with AMD, such as the angiogenesis pathway including FLT1 and VEGFA, the lipid metabolism pathway including ABCA1 and ABCA4, as well as the inflammatory response and complement pathways that include CD59, C3 and C5 (Fig. 1) [refs. 2, 46, 47, 76, 77]. The molecular phenotype in *Abca4*^−/−^ mice, including reduced CD59 and RORA expression, presents much earlier than and likely leads to the clinical phenotype [22, 37]. Downregulated *RORA* expression in *Abca4*^−/−^ mice impacts the pathways required for retinal function and homeostasis, leading to vision loss [2, 46, 47, 76]. The impact of these studies is that treatment with *RORA* before manifestation of the clinical phenotype potentially resets *RORA*-regulated pathways, including the inflammatory response pathway by restoring CD59 expression, thereby preventing disease.

In summary, this study was performed to evaluate efficacy of *RORA* as a modifier gene therapy for macular degeneration. AAV5-*hRORA* rescues STGD and dry AMD-like retinal degeneration, and improves retinal function and molecular outcomes in *Abca4*^−/−^ mice treated at the early to intermediate stage of disease. Interestingly, AAV5-*hRORA* treatment attenuated accumulation of both A2E, the phototransduction waste product that damages RPE cells, as well as APP, the precursor to amyloid-β implicated in pathogenesis in retinal and neurodegenerative diseases [60, 63, 78]. This attenuation, combined with restored expression of the MAC inhibitor, CD59, likely leads to the observed improvement in photoreceptor function. Importantly, no overt evidence of toxicity or abnormal structural changes in the retinas of treated mice was observed. Future studies will examine the longitudinal effect of AAV5-*hRORA* treatment on late-stage retinal degeneration in *Abca4*^−/−^ mice. AMD disease disrupts numerous pathways essential for retinal homeostasis, including inflammation, oxidative stress, lipid metabolism and the complement pathway, which *RORA* regulates [2, 46, 47, 76]. Rescue of retinal degeneration in *Abca4*^−/−^ mice demonstrates the efficacy of *RORA* at resetting multiple disease pathways. Importantly, these studies demonstrate the effectiveness of *RORA* in treating both early-onset STGD and late-onset dry AMD, as mutations in *ABCA4* are observed in both diseases [30–32, 39]. Overall, the data demonstrates that AAV5-*hRORA* has the potential to be a powerfully effective one-time curative modifier gene therapy. *RORA* could be a broad-spectrum treatment for multiple forms of macular degeneration [45]. Two clinical trials are currently ongoing that evaluate the efficacy of *RORA* as a modifier gene therapy in different forms of macular degeneration, including dry AMD (NCT06018558) and STGD (NCT05956626).

### Supplementary information

Supplementary Figure Legends

Figure S1.

Figure S2.

## Data Availability

All data used in this paper has been presented in the figures.
