# Supplementary material for: Retinoic acid related orphan receptor α is a genetic modifier that rescues retinal degeneration in a mouse model of Stargardt disease and Dry AMD
Source: Gene Ther. 2024 May 16;31(7-8):413–21. doi: 10.1038/s41434-024-00455-z (PMC11257945; doi:10.1038/s41434-024-00455-z)
Supplement: Supplementary file 1 — Supplementary Figure Legends [file 41434_2024_455_MOESM1_ESM.docx]

**Supplementary Figure Legends**

**Figure S1. AAV5-*hRORA*-GFP is expressed throughout the fundus.** Blue light autofluorescence (BAF) was observed to be distributed throughout the fundus of B6 animals given a subretinal injection of AAV5-*hRORA*-GFP when compared with untreated B6 mice. n=5.

**Figure S2. RORA expression is restored in the retinal layers of AAV5-*hRORA*-treated *Abca4^-/-^* mice.** A) RORA expression was observed in the GCL, INL and the ONL along with the IS/OS and RPE (insets, closed white arrowheads) in the wild type (WT) strain. A reduction in expression of RORA was observed in the IS/OS and RPE (insets) of untreated *Abca4^-/-^* mice compared with WT mice. Treatment with AAV5-*hRORA* showed restoration of RORA expression in the IS/OS and RPE (insets, closed arrowheads) of treated *Abca4^-/-^* mice. Open arrowheads indicate autofluorescence in blood vessels. B) There is a significant increase in mean fluorescence intensity of RORA in the photoreceptor IS/OS region (*p*<0.05 to 0.0001) and the RPE layer (*p*<0.01 to 0.0001) in *Abca4^-/-^* eyes treated with all doses of AAV5-*hRORA* compared with untreated *Abca4^-/-^* eyes. GCL, ganglion cell layer; INL, inner nuclear layer; ONL, outer nuclear layer; IS/OS, inner segments and outer segments; RPE, retinal pigment epithelium. WT, n≥3; *Abca4^-/-^*, n=5. Scale bars 100 µm.
