# Supplementary figures and images for: Retinoic acid related orphan receptor α is a genetic modifier that rescues retinal degeneration in a mouse model of Stargardt disease and Dry AMD
Source: Gene Ther. 2024 May 16;31(7-8):413–21. doi: 10.1038/s41434-024-00455-z (PMC11257945; doi:10.1038/s41434-024-00455-z)

## Slide 1
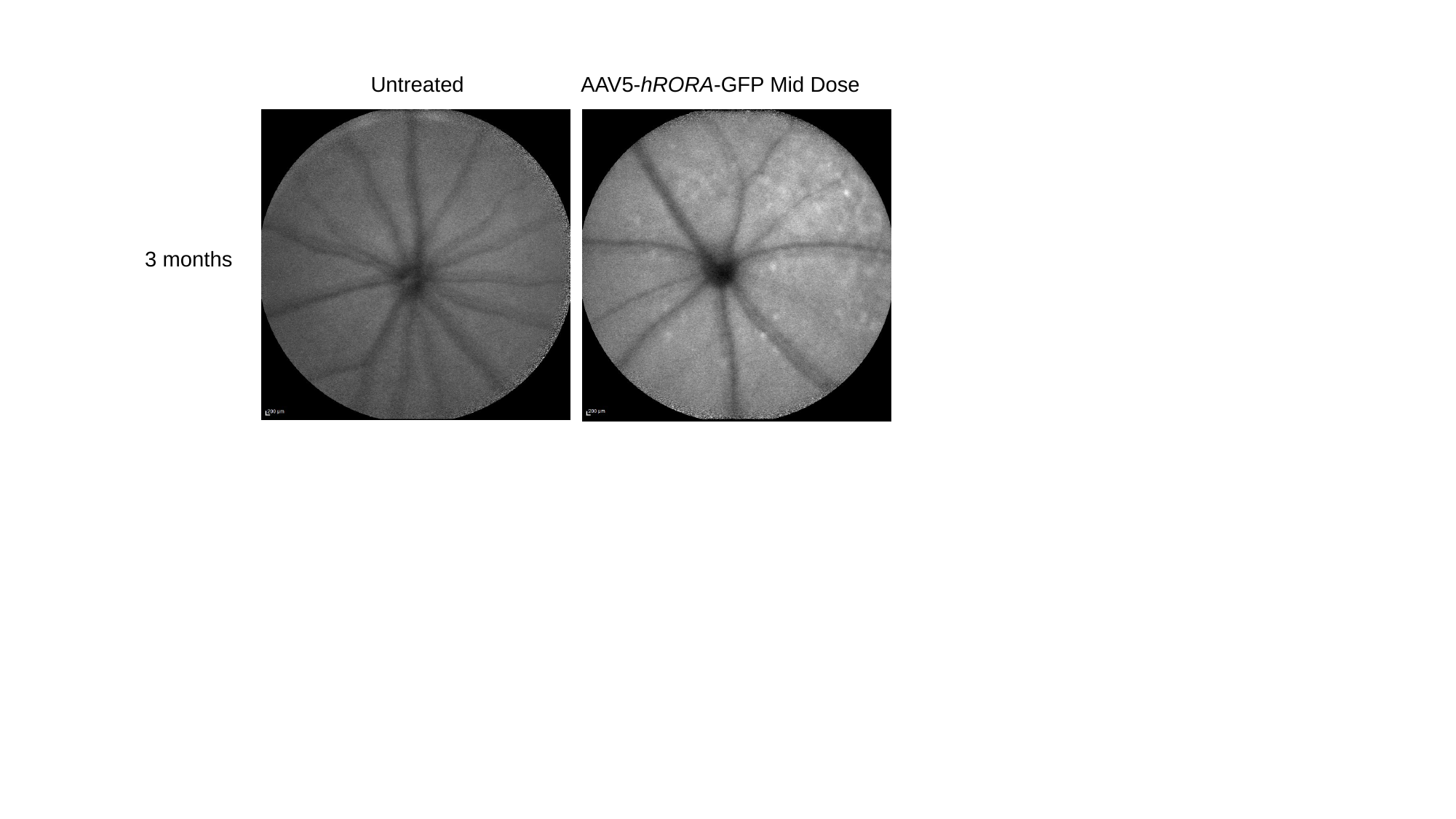

Untreated
AAV5-hRORA-GFP Mid Dose
3 months

Supplement: Supplementary file 2 — Figure S1. [file 41434_2024_455_MOESM2_ESM.pptx]
