# Supplementary material for: Retinoic acid related orphan receptor α is a genetic modifier that rescues retinal degeneration in a mouse model of Stargardt disease and Dry AMD
Source: Gene Ther. 2024 May 16;31(7-8):413–21. doi: 10.1038/s41434-024-00455-z (PMC11257945; doi:10.1038/s41434-024-00455-z)
Supplement: Supplementary file 3 — Figure S2. [file 41434_2024_455_MOESM3_ESM.pptx]

## Slide 1
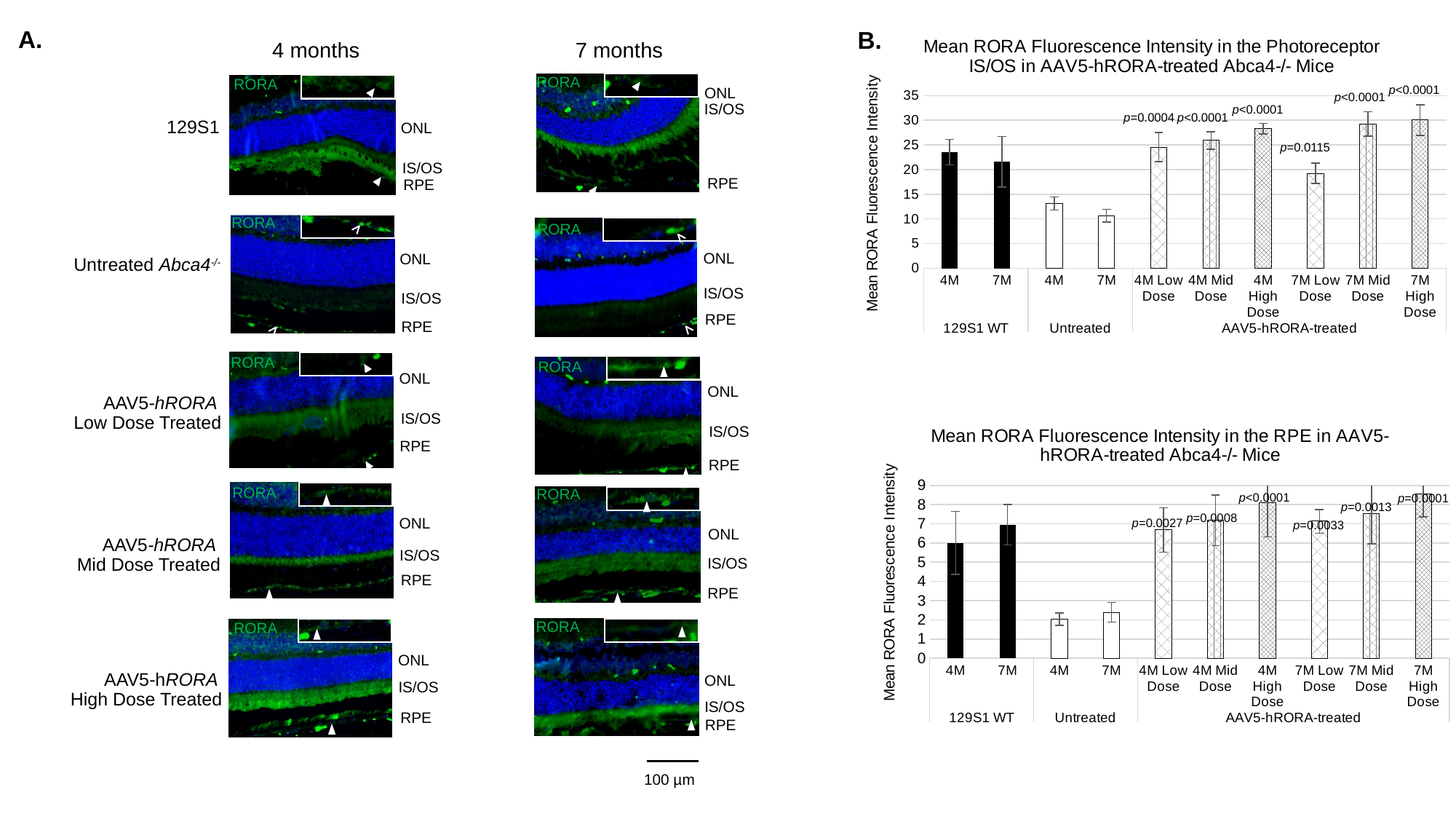

A.
B.
### Chart: Mean RORA Fluorescence Intensity in the Photoreceptor IS/OS in AAV5-hRORA-treated Abca4-/- Mice
| Category | |
|---|---|
| 4M | 23.517514 |
| 7M | 21.603379999999998 |
| 4M | 13.152541333333334 |
| 7M | 10.674100714285716 |
| 4M Low Dose | 24.562214 |
| 4M Mid Dose | 25.917124 |
| 4M High Dose | 28.291592 |
| 7M Low Dose | 19.248058 |
| 7M Mid Dose | 29.262984 |
| 7M High Dose | 30.042558000000003 |4 months
7 months
RORA
RORA
p<0.0001
ONL
p<0.0001
IS/OS
p<0.0001
p<0.0001
p=0.0004
129S1
ONL
p=0.0115
IS/OS
RPE
RPE
RORA
RORA
Untreated Abca4-/-
ONL
ONL
IS/OS
IS/OS
RPE
RPE
RORA
RORA
ONL
ONL
AAV5-hRORA
Low Dose Treated
IS/OS
### Chart: Mean RORA Fluorescence Intensity in the RPE in AAV5-hRORA-treated Abca4-/- Mice
| Category | |
|---|---|
| 4M | 6.0000325 |
| 7M | 6.949916666666667 |
| 4M | 2.031386923076923 |
| 7M | 2.388543 |
| 4M Low Dose | 6.681828 |
| 4M Mid Dose | 7.180134 |
| 4M High Dose | 8.085888 |
| 7M Low Dose | 7.133217999999999 |
| 7M Mid Dose | 7.529368 |
| 7M High Dose | 8.559722 |IS/OS
RPE
RPE
RORA
RORA
p<0.0001
p=0.0001
p=0.0013
p=0.0008
ONL
p=0.0027
p=0.0033
ONL
AAV5-hRORA
Mid Dose Treated
IS/OS
IS/OS
RPE
RPE
RORA
RORA
ONL
ONL
AAV5-hRORA
High Dose Treated
IS/OS
IS/OS
RPE
RPE
100 µm
